# Anti-edema effect of *Aloe vera* leaf extract following traumatic brain injury: Role of pro-inflammatory cytokines

**DOI:** 10.22038/AJP.2021.17426

**Published:** 2021

**Authors:** Marzieh Shahryari, Bahram Bibak, Mohammad Khaksari, Zakieh Keshavarzi, Neda Salmani, Sara Shirazpour, Fatemeh Alimahdi

**Affiliations:** 1 *Neuroscience Research Center, Institute of Neuropharmacology, Kerman University of Medical Sciences, Kerman, Iran*; 2 *Natural Products and Medicinal Plants Research Center, North Khorasan University of Medical Sciences, Bojnurd, Iran*; 3 *Department of Physiology and Pharmacology, School of Medicine, North Khorasan University of Medical Sciences, Bojnurd, Iran*; 4 *Endocrinology and Metabolism Research Center, Institute of Basic and Clinical Physiology Sciences, Kerman University of Medical Sciences, Kerman, Iran *; 5 *Department of Psychology, Genetic Institute, Islamic Azad University- Zarand Branch, Kerman, Iran*; 6 *Physiology Research Center, Institute of Neuropharmacology, Kerman University of Medical Sciences, Kerman, Iran*

**Keywords:** Aloe vera, Traumatic brain injury, Brain edema, IL-1β, TNF-α, TGF-β

## Abstract

**Objective:**

Based on anti-inflammatory effects of *Aloe vera*, the effect of aqueous extract of this plant on brain edema and changes in some pro-inflammatory cytokines was investigated after traumatic brain injury (TBI).

**Materials and Methods:**

In this study, adult male Wistar rats were divided into 5 groups: Sham, TBI, vehicle (Veh), and low dose (LA) and high dose (HA) *Aloe vera*. The vehicle and aqueous extract of *Aloe vera* were injected intraperitoneally 30 min after induction of diffuse TBI by Marmarou’s method. Brain edema (brain water content), and transforming growth factor beta (TGF-β), tumor necrosis factor alpha (TNFα), interleukin 6 (IL-6) and IL-1β levels in serum and brain were measured 24 hr after TBI induction.

**Results:**

Increased brain edema by TBI was reduced by both LA and HA (p<0.01 and p<0.05, respectively). IL-6 increased in the brain of TBI group compared to sham, and which was inhibited by both *Aloe vera* doses compared to Veh (p<0.001). The differences in the IL-6 serum levels among Veh, LA and HA groups were not significant. Increases in serum and brain IL-1β levels were reduced only in the HA group (p<0.001). Although only in the brain, TNF-α level increased after trauma, but both LA and HA inhibited it in a dose-dependent manner (p<0.01 and p<0.05, respectively) . The amount of TGF-β in the brain was reduced by both doses of the extract (p<0.001).

**Conclusion:**

These results indicated that *Aloe vera* has a neuroprotective effect induced by reducing brain edema. The probable mechanism particularly for HA is decreasing levels of pro-inflammatory cytokines such as TGF-β, TNF-α, IL-6 and IL-1β.

## Introduction

Traumatic brain injury (TBI) is one of the most common causes of mortality and disability worldwide (Hyder et al., 2007 ▶). Diffuse axonal injury as a common type of TBI, accounts for nearly half of the severe TBIs (Hyder et al., 2007 ▶). Current estimates show that in the United States, between 2.5 and 6.6 million people live with the consequences of TBI. Many of these TBI are caused by traffic collisions and virtually no effective and approved treatment has been available so far (Valkonen et al., 2004 ▶). In Iran, there are 21,000 traffic collisions per year and approximately 50 to 60% of these collisions lead to death because of TBI (Maas et al., 2008). 

Brain edema leading to an expansion of brain volume, has a crucial impact on morbidity and mortality following TBI (Khaksari et al., 2013 ▶). Brain edema after TBI is accompanied by a series of complicated cytotoxic events and vascular leakage due to the breakdown of the blood-brain barrier (BBB) and it is one of the reasons for delayed brain death following TBI, which is characterized clinically as an increase in the intracranial pressure (ICP) )Rancan et al., 2001 ▶).

Pro-inflammatory cytokines have been reported to cause inflammation and neuronal death following cerebral ischemia which explains the attempts to reduce inflammation or brain edema after the trauma by making changes in the release of pro-inflammatory cytokines (Khaksari et al., 2014 ▶). 

*Aloe vera* belongs to the Asphodelaceae family, which grows in warm and dry environment (Devaraj et al., 2011 ▶). *Aloe vera* has various biological properties including antioxidant (Benedí et al., 2004 ▶) and anti-inflammation (Langmead et al., 2007 ▶) activities and has been shown to reduce the pro-inflammatory cytokines transforming growth factor beta (TGF-β), tumor necrosis factor alpha (TNFα), interleukin 6 (IL-6) and IL-β (Yun et al., 2009 ▶).

Given that the brain edema and neuroinflammation are the main factor for disorders induced by TBI, lack of specific drug for the treatment of brain edema and the importance and recommendation of the use of medicinal herbs, and based on the anti-inflammatory effects of *Aloe vera* shown by previous studies, in the present study, we investigated the effects of aqueous extract of this plant on brain edema and changes in serum and brain levels of some pro-inflammatory cytokines after TBI.

## Materials and Methods

**Animals**

It this study, 70 adult male Wistar rats weighing 200-250 g were used. Animals were kept at a temperature of 20 to 22ºC under 12-hr light- dark cycles in the animal house of the University of Medical Sciences of Kerman. The rats had free access to food and water. This study was conducted in accordance with regulations of Ethics Committee of Kerman University of Medical Sciences No. 95\27.

**Experiment protocol**

Animals were divided into 5 groups: 1. Sham: the animals underwent false brain trauma under anesthesia, but did not receive extract or vehicle. 2. TBI: in this group, animals were anesthetized using Ketamine (2 mg/kg) and xylolin (1 mg/kg) and underwent brain injury induction. 3. Vehicle: brain injury was induced, and animals received an equal volume of vehicle (distilled water) intraperitoneally (IP). 4. Low dose *Aloe vera* (LA): animals in this group received 200 mg/kg of aqueous Aloe vera extract only once (30 min after TBI) IP (Devaraj et al., 2011 ▶) 5. High dose *Aloe vera* (HA): rats in this group were injected with 400 mg/kg of aqueous *Aloe vera* extract only once (30 min after TBI) IP (Devaraj et al., 2011 ▶). Each of the above groups was divided into two subgroups of 7 rats. In one subgroup, brain water content (BWC) was measured 24 hr after TBI and in the other subgroup, cytokine levels were measured 24 hr after TBI in the brain and serum. 

Young leaves of *Aloe vera* were purchased from an herbal shop in Kerman province. Then, the *Aloe vera* leaves were washed in cold water and the bulged parts on leaves were cut and discarded using a knife; finally, the leaves were sliced and 200 g of sliced *Aloe vera* and 100 ml distilled water were mixed in an electric blender (Pars, Iran) for 3 min, and 200% (w/v) extract was obtained.

**Induction of diffuse traumatic brain injury (TBI)**

The Marmarou method was employed to induce the diffuse brain trauma by a device made in the physiology department of Kerman University of Medical Sciences. To induce the TBI, first a steel plate with a diameter of 10 mm and a thickness of 3 mm was fixed along the coronal line between the Bregma and the lambda of the skull of the animal. Then, a 300-g weight was freely dropped from a distance of 2 meters on the head of the animal. In the next step, the animal was immediately restored and the plate was removed from the head of the animal. Finally, the resulted incision was stitched and the animal was returned to the cage (Khaksari et al., 2013 ▶; Khaksari et al., 2015 ▶).

**Measuring brain edema**

The Brain Water Content (BWC) was measured to assess brain edema. To this end, the brain tissue from the anesthetized animal was removed and measured 24 hr after TBI to obtain the wet weight of the brain. This tissue was then heated at 100°C in dry heat oven (Memmert, Germany) for 48 hr and was measured again to obtain the dry weight of the brain. Finally, using the following formula, the brain water was calculated as an index of edema (Guo et al., 2006 ▶).

Percentage of brain water content = (wet weight-dry weight) ×$\frac{100}{\mathrm{wet weight}}$

**Measuring concentration of cytokines in serum**

All the animals were anesthetized with ketamine and xylolin 24 hr after TBI. Then, blood samples were collected from the rats to separate the serum using a centrifuge (G-6B Centrifuge, France). Finally, the samples were measured by ELISA kits (Eastibiopharm, USA), specially produced for measuring TGF-β, TNF-α, IL-6 and IL-1β according to the kit protocol (Taupin et al., 1993 ▶).

**Measuring concentration of cytokines in the brain**

Twenty-four hours after TBI, the brains of anesthetized animals were extracted and frozen by liquid nitrogen. The homogenization of the brains was done by a homogenizer (Heelscher, Germany). To homogenize, 500 mg of each brain was mixed with 2 ml of buffer (pH 7.2) containing 0.5% Triton 100-x, 50 mmol of Tris, 150 mmol of NaCl, and protease inhibitor cocktail (Roche Germany). The homogenized solutions were then centrifuged using a refrigerated centrifuge (Rotina, Germany) at 4000 g at 2°C for 15 min and the top parts of the solutions were used to measure cytokines. The prepared samples were measured using special kits for measuring TGF- β, TNF-α, IL-6 and IL-1β using ELISA method according to the kits’ protocol (Taupin et al., 1993 ▶).

**Statistical analysis**

The results are reported as Mean±SEM. Normal distribution of data was estimated using Shapiro–Wilk test of normality.

Variables in the experimental groups were compared by one-way ANOVA or independent t-test. The Tukey LSD test was used for the ANOVA post-hoc analysis. Percentage of changes in the rats of each group was calculated by the equation: 100 percent × [(data of TBI group − data of vehicle group)/data of vehicle group]. Software SPSS-20 was used for statistical analysis and p-values of less than 0.05 were considered to be statistically significant. 

## Results

**The high and low doses of** ***Aloe vera*** **attenuate the brain edema**

**Figure 1:**
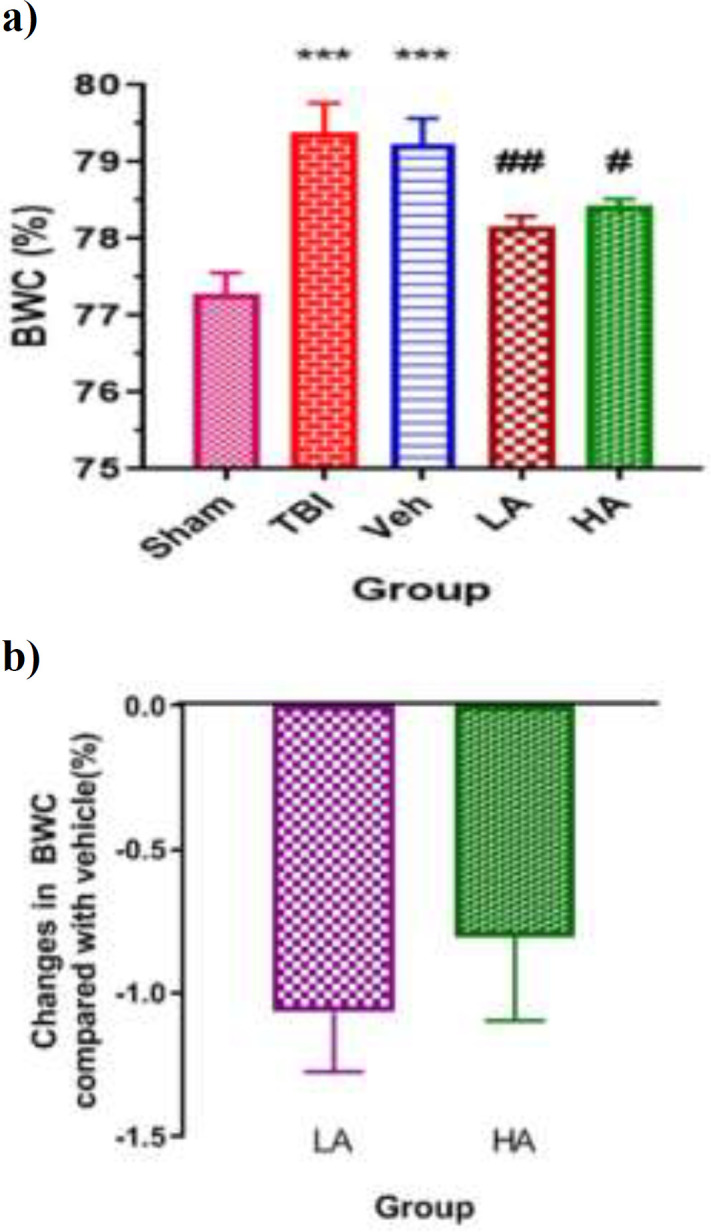
The effect of *Aloe vera* on brain water content) BWC) after TBI. Data are expressed as mean±S.E.M. of n=7 rats/group (a), and changes in BWC (b). ***p<0.001, vs. Sham.^ #^p<0.05, and ^##^p<0.01 vs. Veh. There were no significant differences among the groups. TBI: Traumatic Brain Injury; LA: Low dose of *Aloe vera*. HA: High dose of Aloe vera; and Veh: Vehicle

Figure 1 (a) shows the changes in the BWC in different groups. The BWC in the TBI (79.39±0.38%) and Veh groups (79.23±0.34%) were significantly (p<0.001) more than that in the sham group (77.28±0.28%). The BWC in the LA (78.16±0.14%) and HA (78.44±0.99%) groups was less than the BWC in the Veh group (p<0.01 and p<0.05, respectively). Figure 1 (b) shows the percentages of changes in BWC in the LA and HA groups in comparison with the Veh group. The percentages of reduction in the LA group was 1.07±0.20% and in the HA group was 0.80±0.29%, which were not significantly different from each other. These results indicated that both doses had neuroprotective effects.

**IL-1β in brain and serum was decreased by high dose of** ***Aloe vera***

**Figure 2.**
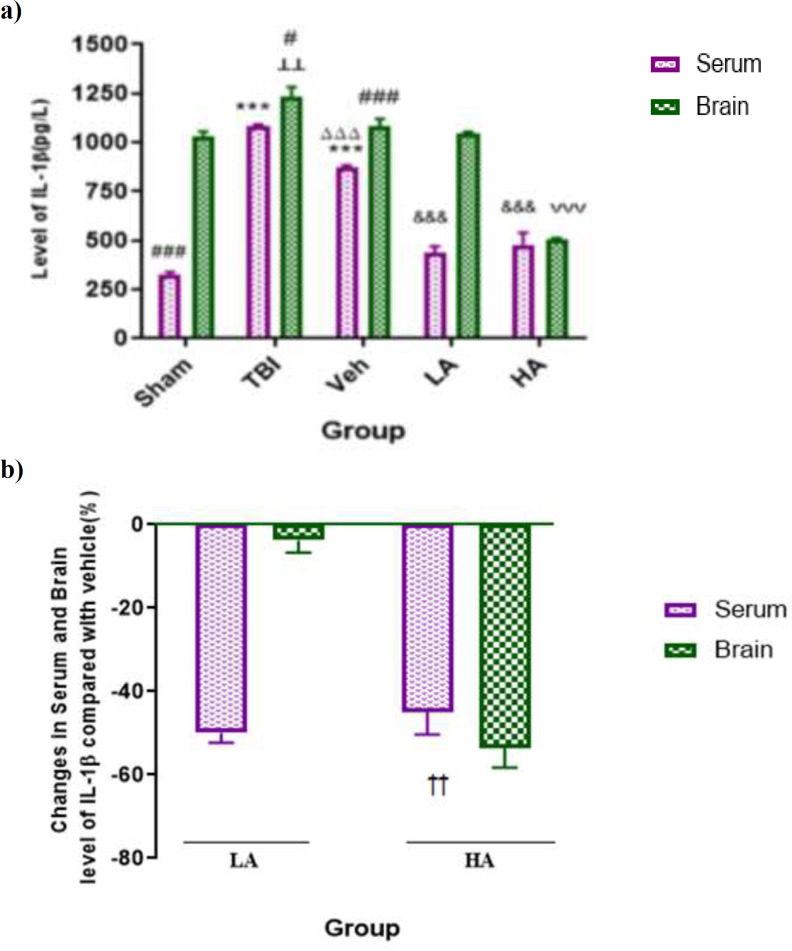
Comparison of IL-1β levels in serum and brain (pg/L) among different groups. Data are expressed as mean±S.E.M. of n=7 rats/group (a), and changes in BWC (b). ***p<0.001, vs. sham serum. ^┴┴^p<0.01, vs. Sham brain. ^ ΔΔΔ^p<0.001, vs. TBI.^ #^p<0.05, and ^###^p<0.001 significant differences between serum and brain level. ^&^^&&^p<0.001, vs. Veh serum.^ ˅˅˅^^,^ p<0.001, vs. Veh, and LA brain.^ ††^p<0.01 vs. LA brain in panel (b). TBI: Traumatic Brain Injury; LA: Low dose of Aloe vera; HA: High dose of Aloe vera and Veh: Vehicle

 In Figure 2 (a), the serum and brain levels of IL-1β in all groups are shown. The serum level of IL-1β in the TBI (1082.8±8 pg/L), and Veh (870.1±13 pg/L) groups were significantly (p<0.001) higher than that in the sham group (323.8±17 pg/L). The serum level of this cytokine in the Veh group was lower than the one in the TBI group (p<0.001). Also, the brain IL-1β level in the TBI group (1230±21 pg/L) was greater than that in the sham group (1020±27 pg/L, p<0.01). In addition, the brain levels of this cytokine in the Sham, TBI and Veh groups were higher than its serum levels in these groups (p<0.001, p<0.05, and p<0.001, respectively). 

Serum IL-1β levels in the LA (36.33±33.09 pg/L) and HA groups (61.61±71.7 pg/L) /478) were less than that in the Veh group (p<0.001). In addition, the brain level of this cytokine in the HA group (507.68±3.25 pg/L) was lower than that of the Veh and LA groups (p<0.001).

Figure 2 (b) illustrates changes in percentage of IL-1β level compared with Veh; the HA group had significantly reduced IL-1β levels in brain (53.66±4.67%) compared with the LA group (p<0.001). Reduction in IL-1β brain level in the HA group was higher than the LA group. There was no significant difference between changes in percentage of the serum and brain levels of IL-1β​​ in the HA group.

**TNF-α levels in brain and serum samples was more drastically decreased by HA dose**

**Figure 3:**
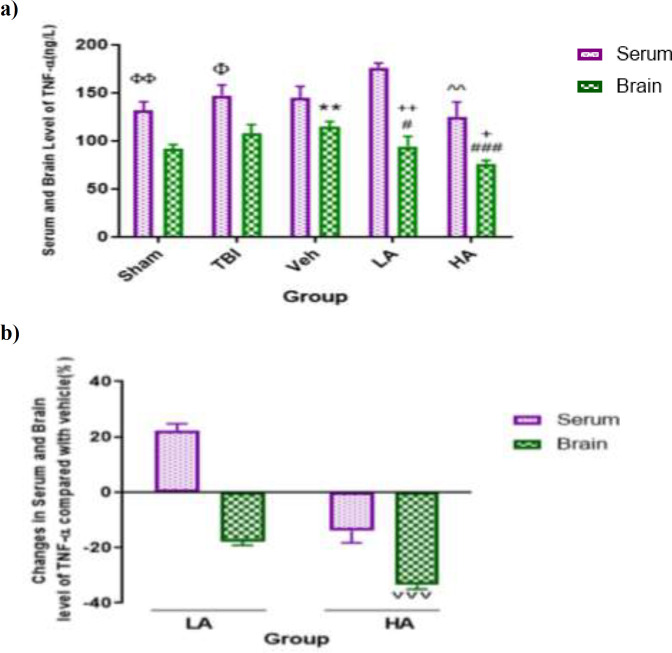
Comparison of TNF-α level in serum and brain (pg/L) among different groups. Data are expressed as mean±S.E.M. of n=7 rats/group (a), and changes in BWC (b). **p<0.01, vs. sham brain, Φp<0.05, and ΦΦp<0.01 significant different between serum and brain level. #p<0.05, and ###p<0.001 vs. Veh brain. ^^p<0.01 vs. LA serum. +p<0.05, and ++p<0.01 significant different between the serum and brain levels. ˅˅˅ p<0.001 vs. LA brain in panel (b).TBI: Traumatic Brain Injury; LA: Low dose of *Aloe vera*; HA: High dose of *Aloe vera*; and Veh: Vehicle

The brain and serum levels of TNF-α in different groups are shown in Figure 3 (a). There was no significant difference among the sham (131.69±8.9 ng/L), TBI, and Veh groups in terms of serum TNF-α level. But, the brain level of this cytokine in the Veh group (114.75±5.42 ng/L) was greater than that in the sham group (91.71±4.59 ng/L, (p<0.01). The serum levels of TNF-α in the sham and TBI groups were higher than its brain levels in these groups (p<0.01, p<0.05, respectively).

The serum level of TNF-α in the LA, and HA groups was not significantly different from the Veh group but the serum level of this cytokine in the HA group (125.16±15.25 ng/L) was significantly lower than that in the LA group (p<0.01). Also, the brain levels of TNF-α in the LA (94.4±5.55 ng/L) and HA groups (76.11±3.39 ng/L) were lower than that in the Veh group (p<0.05, and p<0.001 respectively). In addition, brain level of TNF-α in the HA group was lower than that in the LA group (p<0.05). Brain levels of TNF-α in the LA and HA groups were lower than its serum levels in these groups (p<0.01 and p<0.05, respectively). Figure 3 (b) reveals the percentages of changes in TNF-α in the LA and HA groups compared with the Veh group. Percentage of decrease in brain level of TNF-α in the HA group was higher than that in the LA group (p<0.001). Reduction in TNF-α brain level in HA group was higher than that of the LA group.

**Both doses of** ***Aloe vera*** **doses decreased the level of IL-6**

**Figure 4:**
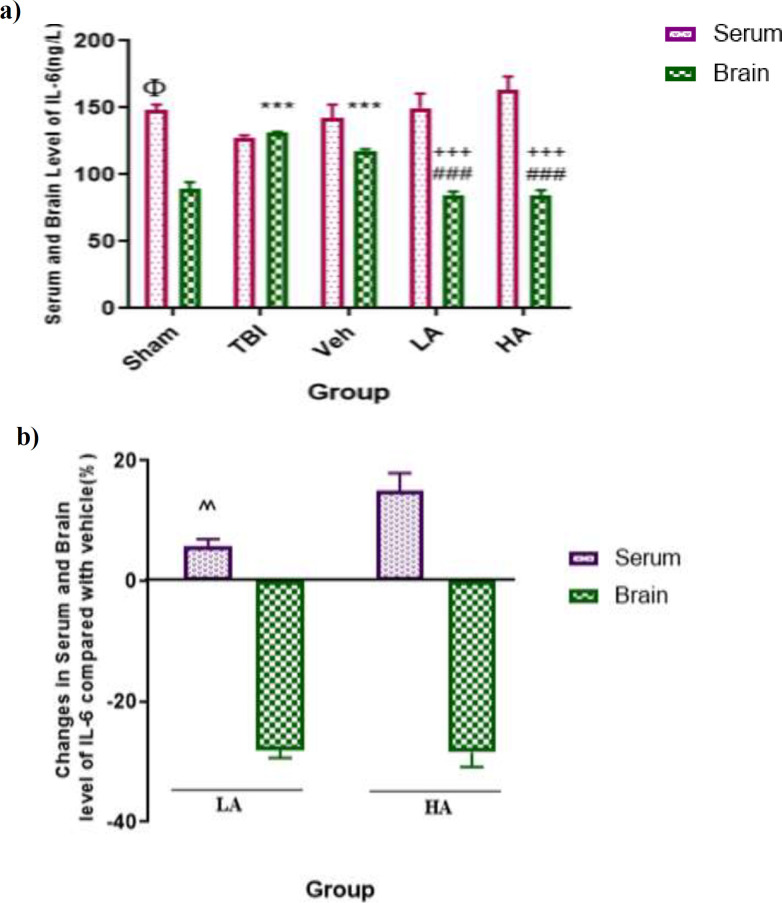
Comparison of IL-6 level in serum and brain (pg/L) among different groups. Data are expressed as mean±S.E.M. of n=7 rats/group (a), and changes in BWC (b). ***p<0.001 vs. sham brain. ^Φ^p<0.05 vs. sham brain,^ ###^p<0.001 vs. Veh brain.^ +++^p<0.001 significant difference between serum and brain level. ^^^^p<0.01 vs. HA serum in panel (b). TBI: Traumatic Brain Injury; LA: Low dose of *Aloe vera*; HA: High dose of *Aloe vera*; and Veh: Vehicle

In Figure 4 (a), the serum levels of IL-6 in all groups are shown. The differences between the serum levels of IL-6 in the sham (148.39±3.92 ng/L), TBI (127.74±1.65 ng/L) and Veh (142.08±10.49 ng/L) groups were not significant. Brain IL-6 levels in the TBI (131.52±0.54 ng/L) and Veh groups (117.68±1.43 ng/L) had significant differences with that in the sham group (89.11±5.21 ng/L, p<0.001). Also, serum IL-6 levels were significantly higher than its brain level in the sham group (p<0.01). Differences in the serum levels of IL-6 among the Veh, LA (149.69±10.63 ng/L) and HA (163.3±9.19 ng/L) groups were not significant. Brain IL-6 level in the LA (84.66±2.63 ng/L) and HA groups (84.14±24.4 ng/L) were significantly lower than that in the Veh group (p<0.001).

Figure 4 (b) shows the changes in percentage of serum and brain levels of IL-6 in the LA and HA groups compared with the Veh group. The percentage of increase in serum IL-6 level in the LA group was lower than that in the HA group (p<0.01). There was no significant difference between the LA and HA groups in terms of percentage of decrease in IL-6 level. This results indicated that both doses had a similar effect.

**High and low doses of** ***Aloe vera*** **decreased brain TGF-β level**

**Figure 5:**
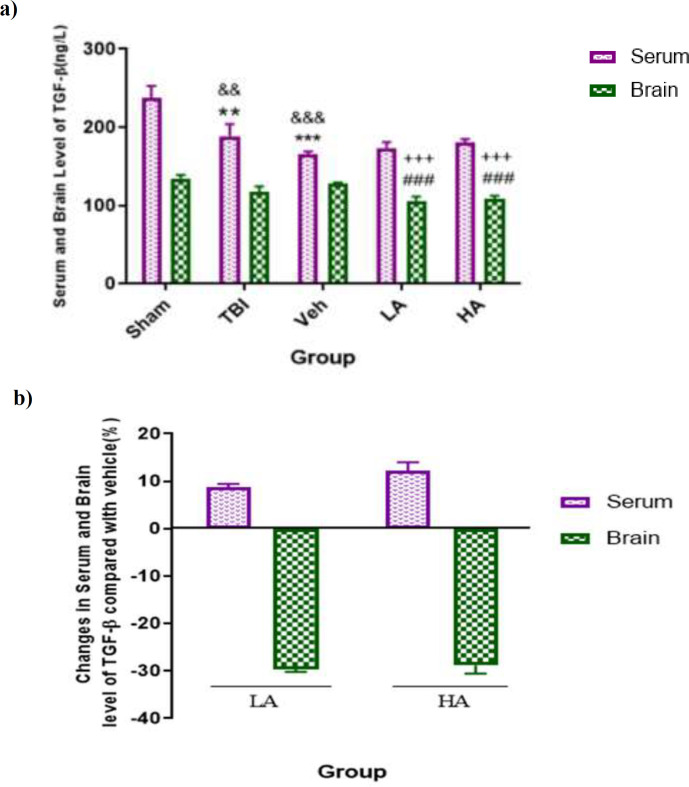
Comparison of TGF-β level in serum and brain (pg/L) among different groups. Data are expressed as mean±S.E.M. of n=7 rats/group (a), and changes in BWC (b). **p<0.01, and ***p<0.001 vs. sham serum. ^&&^p<0.01, and ^&&&^p<0.001 significant difference between serum and brain levels.,^ ###^p<0.001 vs. Veh brain.^ +++^p<0.001 significant difference between serum and brain level. There were no significant differences among the groups. TBI: Traumatic Brain Injury; LA: Low dose of *Aloe vera*; HA: High dose of *Aloe vera*; and Veh: Vehicle

In Figure 5 (a), brain and serum level of TGF-β in different groups are shown. The TGF-β serum level in the Veh (165.37±3.69 ng/L) and TBI groups (188.97±15.23 ng/L) was lower than that in the sham group (p<0.01, and p<0.001, respectively). Also, serum level of TGF-β in the sham, Veh and TBI groups was higher than the brain levels of this cytokine (p<0.001, p<0.01 and p<0.001, respectively). There were no significant differences in serum TGF-β level in the Veh, LA (179.87±7.20 ng/L) and HA groups (180.32±4.84 ng/L). 

On the other hand, the brain levels of TGF-β in the LA (91.21±3.27 ng/L) and HA groups (90.07±1.89 ng/L) were lower than that in the Veh group (p<0.001). This means that the brain level of TGF-β was only reduced by both doses of *Aloe vera*. In addition, brain TGF-β levels in the LA and HA groups were lower than the serum levels of TGF-β in these groups (p<0.001). These results indicated that variation similar to TNF-α level, because the TNF-α serum level was higher than brain level. Figure 5 (b) shows the percentages of changes in TGF-β in the LA and HA groups in comparison with the Veh group. There were no significant differences between the LA and HA groups in brain, and serum levels of this cytokine.

## Discussion

In this study, the effect of aqueous extract of *Aloe vera* on brain edema and some inflammatory cytokines in the brain and serum in response to TBI, was investigated. The main findings of this study were: 1. the brain edema after TBI was reduced by high and low doses of *Aloe vera*; 2. Increases in IL-6 in the brain of animals with TBI were inhibited by both doses of *Aloe vera*. 3. The increased IL-1β level in serum after TBI was decreased by both doses, but the IL-1β level in brain was only decreased by the high dose of *Aloe vera*. 4. The increased TNF- α and TGF-β brain levels after trauma were decreased by both doses, although none of the doses affected serum levels of these cytokines.

In the present study, a significant 2.1% increase in brain water content was observed in the traumatic group, while brain water content in vehicle-treated animals and the trauma group was not significantly different. The results of our previous studies also confirmed this increase in brain edema after TBI in both male (Keshavarzi et al., 2012 ▶) and in female animals (Khaksari et al., 2013 ▶) after TBI. TBI causes secondary and complex injury through multiple mechanisms such as brain edema, BBB breakdown, neuro-inflammation, hypoxia and cell death (Abbas et al., 2014 ▶; Loane et al., 2010 ▶). Both vasogenic and cytotoxic edema play a role in TBI. Vasogenic edema occurs within hours after TBI, while cytotoxic edema develops within a few days (Bramlett et al., 2009 ▶; Barzo et al., 1997 ▶). Nevertheless, the mechanism involved in edema formation is not fully understood. The increase in the brain edema (brain water content) is due to the increase of sodium ion along with water in the brain. This increase of sodium ion can be attributed to the breakdown of BBB (Benigni et al., 2010 ▶), vascular endothelium injury, damage to astrocytes and in some cases the increase in the transfer of vesicles from vascular endothelium (Park et al., 2009 ▶).

Low dose of *Aloe vera* reduced TBI-induced brain edema by 1.07% and high dose by 0.8%, although there was no difference between the two doses. It seems that the *Aloe vera* extract decreases brain edema through the possible mechanism(s): Antiinflammatory activity due to the presence of phenolic compounds or anthraquinone compounds and chromones (Kumar et al., 2007 ▶), possibly by neutralizing the activity of neutrophils leukotriene B4, prevention of the activation and recruitment of inflammatory cells and reduction in pro-inflammatory cytokines, increase in the anti-inflammatory cytokines (Eamlamnam et al., 2006 ▶), reduction in the production of nitric oxide from macrophages (Sarkar et al., 2005 ▶) and reduction in oxidative stress (Shahrokhi et al., 2012 ▶) because of vitamins A, C, and E being present in extract (Brough et al., 2011 ▶; Mofid et al., 2016 ▶). It was found that no study has examined the effect of Aloe vera on brain edema, and only one study reported that *Aloe vera* has no effect on brain edema after gastric ulcer (Keshavarzi et al., 2014 ▶).

In another part of this study, the effect of different doses of *Aloe vera* extract on the brain and serum levels of IL-1β was showed that the IL-1β level increased after cerebral trauma in the brain and serum. A low dose (50%) and a high dose (45%) of the extract reduced the level of IL-1β in the serum, but high dose of the extract reduced the level of IL-1β in the brain. The effect of high dose of the extract on IL-1β level was higher than the low dose. Another comparison in this study was made between the brain and serum levels of IL-1β in all the groups. The level of IL-1β in the brain in all groups except the high dose group, was higher than its serum level.

IL-1β is a pro-inflammatory cytokine which plays a role in the breakdown of BBB and formation of edema (Jones et al., 2004 ▶) through releasing phospholipase A2 and prostaglandin, activation of cyclooxygenase 2, chemokines and matrix metalloproteinases (MMP) (Rothwell et al., 2003 ▶), and regulating the release of other cytokines like IL-6, and TNF-α (Jones et al., 2004 ▶). The results of this study are in line with other studies in this regard. For instance, Haykata et al. reported that IL-1β increases after TBI in blood and serum (Hayakata et al., 2004 ▶). Stain et al. reported increased IL-1β in the cerebrospinal fluid (CSF) and serum of patients (Stein et al., 2011 ▶). Additionally, in patients with severe brain injury, an increase in IL-1β concentration in the CSF is associated with poor neurological complications and increased intracranial pressure (ICP) (Intiso et al., 2004 ▶). Since, IL-1β level was increased both in brain and serum after TBI, it is likely that the origin of IL-1β was brain cells because it has been reported that microglia and astrocytes produce IL-1β after brain injury (Woodroofe et al., 1991 ▶).

This study was the first report on the effects of *Aloe vera* extract on changes in cytokines levels after TBI, and there is no similar study for comparing the effects of *Aloe vera* after TBI. However, there are some studies pointing to the anti-inflammatory effect of *Aloe vera*. *Aloe vera* reduces the expression of inflammatory cytokines, especially IL-1β and TNF-α in rats with colitis which is probably because of the presence of compounds such as aloin and aloesin in the plant (Park et al., 2011 ▶). Yun et al. showed that *Aloe vera* gel reduces inflammation by microbial infection, and decreasing the IL-1β levels (Yun et al., 2009 ▶). The use of *Aloe vera* gel powder considerably reduced the production of inflammatory cytokines induced by bacteria (Habeeb et al., 2007 ▶). There is no conflicting report on the effect of Aloe vera on IL-1β.

In the current study, we also observed that unlike IL-1β which increased after brain injury, TNF-α did not increase in brain. Since the vehicle increased this cytokine in the brain, the level of this inflammatory factor was reduced by high doses (34%) and low doses (18%).

TNF-α is a multi-function pro-inflammatory cytokine. Although this protein is mainly produced by macrophages, glial cells, endothelial cells, neutrophils and lymphocytes, neuronal production has also been reported (Habeeb et al., 2007 ▶). TNF-α activities are as follows (Na'ama et al., 2007 ▶): activating neutrophils and macrophages in releasing oxidants, proteases, arachidonic acid metabolites, and production of other cytokines such as IL-1β and IL-6, and increasing vascular endothelial permeability (Kim et al., 1992 ▶). Similar to the present study, it has been shown that TNF-α expression was increased during the first hours after trauma and high levels of this cytokine during the first hours after injury cause subsequent damages including BBB breakdown and brain edema in animal models (Goodman et al., 1990 ▶).

In our study, it was found that TNF-α increases in the TBI group receiving vehicle which is in line with other studies reporting increase of this cytokine in the brain after brain injury. Goodman et al. reported that after TBI, TNF-α levels increased in both serum and CSF (Goodman et al., 1990 ▶). Previous study showed that TNF-α level increased in TBI patients (Dalgard et al., 2012 ▶). It has also been reported that TNF-α reflects the severity of injury (Knoblach et al., 1999). Stain et al. reported that there is an association between cytokine levels and changes in intracranial pressure (ICP) and cerebral perfusion pressure (CPP). Therefore, increases in serum and brain concentration are consistent with increases in ICP and decreases in CPP (Stein et al., 2011 ▶). Since there are no studies investigating the effects of Aloe vera on the TNF-α level after TBI, the effects of Aloe vera on the TNF-α level are discussed with reference to studies on the effects of Aloe vera on TNF-α in non-TBI cases. Aloe vera reduced the inflammation through microbial infection, lowering TNF-α level (Yun et al., 2009 ▶), decreasing leukocyte adhesion and TNF-α levels in rats infected with *Helicobacter pylori* (Prabjone et al., 2006 ▶), and improving the healing of the peptic ulcer by reducing the TNF-α level (Eamlamnam et al., 2006 ▶) in non-TBI cases. In the present study, it was found that unlike IL-1β, the brain level of TNF-α was lower than its serum level which contradicts with the results of the studies reporting higher levels of TNF-α in CSF (Woodcock et al., 2013 ▶). 

The present study also showed that the brain level of IL-6 increased after TBI, although its serum level did not change. These results are consistent with the results of previous studies (Hang et al., 2004 ▶; Soltani et al., 2016 ▶). IL-6 Knoblach is one of the important factors in the development of acute TBI responses. Studies have shown that the enhancement of IL-6 caused neuroinflammation and brain damage (Berger et al., 2009 ▶). This interleukin has a dual role in inflammation. It has been shown that although IL-6 plays a major role in reacting to acute inflammation and is produced by endothelial cells and neurons, it has neuroprotective role by inhibiting TNF-α, inducing IL-1ra and stimulating the production of nerve growth factor (NGF), defending against oxidative stress and glutamate-mediated toxicity, and promoting angiogenesis (Morganti-Kossmann et al., 2010 ▶). The reasons for the difference between the results of the present study and the ones mentioned above could be attributed to the type of trauma, severity of injury and the type of the study.

It was also observed that different doses of *Aloe vera* reduced IL-6 level in the brain. When comparing the levels of this cytokine between the brain and the serum in the *Aloe vera* groups, reduction of the IL-6 was greater in the brain than serum. This reduction in IL-6 by two doses of Aloe Vera is consistent with the of Yun study who showed that *Aloe vera* gel reduced microbial inflammation by decreasing the IL-6 levels (Yun et al., 2009 ▶). It has also been reported that *Aloe vera* gel reduced IL-6 in rats with skin burn (Duansak et al., 2003 ▶). No reports have noted the increase in IL-6 levels by *Aloe vera*. It was observed that the TGF-β level like the TNF-α level did not increase after TBI in the brain although its level in serum decreased in the TBI and vehicle groups. This finding is in line with the result of the study of Sarkaki et al. who reported that the TGF-β levels in the animals’ brains after TBI did not change (Sarkaki et al., 2013 ▶). However, another study suggested that the TGF-β and IL-1β levels were reduced because of the reduction in brain water content (Kovacs et al., 2005 ▶). In addition, Bibak et al. reported that calcium channel blockers are probably responsible for the inhibition of brain edema by reducing this cytokine (Bibak et al., 2007 ▶), but its neuroprotective effect is greater than its negative effect (Gibson et al., 2005 ▶). It was observed that serum level of TGF-β decreased after using vehicle. TGF-β is released after damage to the tissues by degranulation from the platelets in wound site. Since many studies have investigated the neuropathic effects of TGF-β and have reported conflicting findings such as the beneficial effects of progesterone along with the reduction in TGF-β, the reduction of TGF-β after TBI which inhibited the production of IL-1β, TNF-α, and oxygen free radicals may have lowered the inflammation (Habeeb et al., 2007 ▶).

Both high and low doses of Aloe vera extract reduced the brain levels of TGF-β 29% and 29.83% respectively but did not change the levels of the IL-6 in the serum. After comparing the brain and serum levels of this cytokine, it was found that the brain level of TGF-β was lower than its serum level. In contrast to our results, Jafarzadeh et al. reported that the *Aloe vera* extract increased the gene expression of TGF-β in non-TBI cases (e.g. wound skin), (Jafarzadeh et al., 2014 ▶). However, since there is no research on the effect of *Aloe vera* extract on TGF-β after TBI, we cannot be certain that the observed changes in TGF-β level are positive or negative. There is a limitation in the present study due to animal mortality during TBI induction.

The neuroprotective effect of *Aloe vera* could be attributed to its anti-inflammatory effect. Increased levels of brain proinflammatory cytokines such as IL-1β, IL-6, TNF-α and TGF-β after TBI were reduced by both low and high doses of Aloe vera. These results suggest preventive therapeutic potential of *Aloe vera* in TBI. However, further studies are needed to evaluate the effects of *Aloe vera* and its constituents in animal models of TBI and humans.
